# The complex interactions between the cellular and non-cellular components of the brain tumor microenvironmental landscape and their therapeutic implications

**DOI:** 10.3389/fonc.2022.1005069

**Published:** 2022-10-06

**Authors:** Syed M. Faisal, Andrea Comba, Maria L. Varela, Anna E. Argento, Emily Brumley, Clifford Abel, Maria G. Castro, Pedro R. Lowenstein

**Affiliations:** ^1^ Dept. of Neurosurgery, University of Michigan Medical School, Ann Arbor, MI, United States; ^2^ Dept. of Cell and Developmental Biology, University of Michigan Medical School, Ann Arbor, MI, United States; ^3^ Rogel Cancer Center, University of Michigan Medical School, Ann Arbor, MI, United States; ^4^ Dept. of Biomedical Engineering, University of Michigan, Ann Arbor, MI, United States

**Keywords:** GBM, ECM, collagen, 2HG, exosomes, cytokines, cellular biomechanics

## Abstract

Glioblastoma (GBM), an aggressive high-grade glial tumor, is resistant to therapy and has a poor prognosis due to its universal recurrence rate. GBM cells interact with the non-cellular components in the tumor microenvironment (TME), facilitating their rapid growth, evolution, and invasion into the normal brain. Herein we discuss the complexity of the interactions between the cellular and non-cellular components of the TME and advances in the field as a whole. While the stroma of non-central nervous system (CNS) tissues is abundant in fibrillary collagens, laminins, and fibronectin, the normal brain extracellular matrix (ECM) predominantly includes proteoglycans, glycoproteins, and glycosaminoglycans, with fibrillary components typically found only in association with the vasculature. However, recent studies have found that in GBMs, the microenvironment evolves into a more complex array of components, with upregulated collagen gene expression and aligned fibrillary ECM networks. The interactions of glioma cells with the ECM and the degradation of matrix barriers are crucial for both single-cell and collective invasion into neighboring brain tissue. ECM-regulated mechanisms also contribute to immune exclusion, resulting in a major challenge to immunotherapy delivery and efficacy. Glioma cells chemically and physically control the function of their environment, co-opting complex signaling networks for their own benefit, resulting in radio- and chemo-resistance, tumor recurrence, and cancer progression. Targeting these interactions is an attractive strategy for overcoming therapy resistance, and we will discuss recent advances in preclinical studies, current clinical trials, and potential future clinical applications. In this review, we also provide a comprehensive discussion of the complexities of the interconnected cellular and non-cellular components of the microenvironmental landscape of brain tumors to guide the development of safe and effective therapeutic strategies against brain cancer.

## Introduction

Glioblastomas (GBM) are the most aggressive and invasive tumors of the central nervous system. GBM are highly heterogeneous, and the complex tumor cell interactions with the cellular and non-cellular (ECM) TME influence overall tumor progression (1). Aside from malignant cells, tumors are comprised of vascular cells and immune cells (macrophages, microglia, and T, B, and NK cells) -which in gliomas can amount to 50% of the total number of cells in the tumor- in addition to the extracellular matrix (2). The ECM is modified in GBM tumors compared to the normal brain, playing a critical role in tumor migration and invasion (3, 4). In GBM, the ECM consists of higher levels of collagen, fibronectin, laminin, hyaluronic acid, tenascin-C and vitronectin (4, 5) and the exact composition of any individual tumor varies with the stage of tumor growth as the extracellular matrix becomes more remodeled (6, 7).

Unfortunately, there has been very little therapeutic success for glioblastomas and high rates of recurrence. Interestingly, very few clinical trials focus on microenvironmental targets, such as the ECM or immune cell-tumor interactions. For example, macrophages display two different phenotypes, pro-tumoral M2 and anti-tumoral M1, and interactions between pro-tumoral macrophages can alter the mesenchymal characteristics of glioma cells (8–10). Thus, it might be possible to tip the balance towards the anti-tumoral macrophage as a therapeutic strategy. Another unexplored possibility would be to understand in further depth the interactions between immune cells and the extracellular matrix. Immune cells comprise a complex group of T cells, B cells, and NK cells, but in spite of many expectations, clinical trials using immune checkpoint inhibitors have only achieved minor anti-tumor effects (11). In other tumors, it has been shown that manipulating the ECM can alter the capacity of immune cells to enter the tumor mass (12, 13). Thus, the ECM-immune cell interaction remains an unexplored potential target for the treatment of GBM.

Other novel therapeutic strategies now consider the possibilities of targeting the ECM with therapeutic intent (14). As our knowledge of the structure of tumor ECM progresses, novel potential targets are discovered. An intriguing possibility is the direct targeting of structures formed by glioma cells and called oncostreams (15). These structures promote glioma growth and invasion and depend on their structure on collagen produced by tumor cells. As we have shown that eliminating collagen from tumor cells disrupts oncostream structure, we propose that doing so pharmacologically may be a potential novel therapy. Interestingly, similar production of Collagen 1 by tumor cells has recently been shown in pancreatic cancer (16). Though there have not yet been clinical trials in GBM tumors targeting the ECM, such trials have been performed in pancreatic cancer (17, 18). However, in spite of very positive preclinical data, there were no increased survival in patients treated with inhibitors of hyaluronic acid (19).

Targeting the ECM therapeutically carries its own challenges, as it remains unclear which are the cells that produce the ECM. There exists a population of brain fibroblasts, though it is yet poorly understood (20). It is likely that such fibroblasts contribute to the tumor ECM. If they do, it will be important to determine which factors that induce these cells to alter the composition of the ECM as tumors grow. Interestingly, it is now becoming clear that tumor cells themselves contribute to the makeup of the ECM. Tumor cell contributions are thus another potential treatment target. In the case of genetically engineered mouse models of glioma we have recently shown that tumor cells produce Collagen 1 (15), and it has further been shown that breast cancer cells also produce a particular isoform of Collagen 1 (21). As knowledge advances concerning the contributions tumor cells make to tumor ECM, novel therapeutic targets will be identified. The larger themes of tumor-ECM interaction which have been highlighted will now be discussed in detail throughout this review.

## Glioma subtypes and molecular classification

GBMs vary in terms of histologic characteristics, malignancy grade, and molecular changes. The histological WHO classification of gliomas, which previously classified these tumors as being of glial origin, has recently been improved by the addition of the existence and distribution of genetic/epigenetic changes as classification criteria (22–24). Classifying gliomas based on recurrent IDH1 point mutations, which have been linked to gliomagenesis (25, 26), is important because it distinguishes mutant IDH1 gliomas from wild-type-IDH1 (wt-IDH1) gliomas. In contrast to low-grade gliomas (LGG), WHO grade 4 wt-IDH1 high-grade gliomas exhibit more somatic mutations and multiple genomic alterations (22, 27–30). Adult wt-IDH1 gliomas still demonstrate ATRX activity and frequently co-present TP53 and TERTp mutations. Additionally, wt-IDH1 gliomas can have mutations or deletions in the tumor-suppressor genes PTEN and CDKN2A/B, as well as changes to chromosomes 7 and 10. These changes can affect how the RTK-RAS-PI3K signaling cascade is regulated, including EGFR amplification (23, 27).

The majority of diffuse LGG (WHO grade 2) and LGGs that relapse as GBM (WHO grade 4) have IDH1 mutations, typically at arginine 132 (R132H) (29, 31–33). IDH1-R132H catalyzes the formation of 2-hydroxyglutarate, which leads to epigenetic reprogramming of the glioma transcriptome and is associated with a better prognosis in glioma (32, 34–36). A mutant IDH1 glioma subgroup with codeletion of the chromosomal bands 1p and 19q as well as a mutation in the TERT promoter are histologically defined as oligodendrogliomas (37–39). In contrast, mutant IDH1 gliomas without 1p and 19q codeletion are typically P53 and ATRX mutants and they have astrocytic histology with a hypermethylation phenotype (G-CIMP high). The importance of IDH1 mutation status to the clinical fate (better prognosis) of the tumors contributed to the decision to include it in the classification of diffuse gliomas (40, 41). Furthermore, demethylation of CXCR4, TBX18, SP5, and TMEM22 genes is also associated with the initiation and progression of tumors in GBM (42). Analyzing methylation profiles of TCGA data uncovered DNA methylation clusters termed subtypes LGm1 through LGm6, which were associated with molecular glioma subclasses and WHO grades (32). Additionally, methylation of MGMT promoter CpG islands predicts improved response to DNA alkylating drugs (43). In terms of methylation and copy number profile, as well as histological appearance and molecular signature, a novel IDH1-WT GBM methylation subgroup that differs from previously reported molecular subgroups was recently introduced in the classification of glioma (44).

The 2021 WHO classification of gliomas has introduced 4 general groups of diffuse gliomas: adult-type diffuse, pediatric-type diffuse low-grade, pediatric-type diffuse high-grade, and circumscribed astrocytic gliomas. Adult-type diffuse gliomas include astrocytoma with IDH mutation, oligodendroglioma with IDH mutation and 1p/19q codeletion, and glioblastoma with IDH wildtype. IDH-mutant diffuse astrocytomas are now classified 2-4 grade; the designations “anaplastic astrocytoma” and “glioblastoma” have been dropped for IDH-mutant astrocytomas. Furthermore, if an IDH-mutant diffuse astrocytoma has a homozygous deletion of CDKN2A/B, it is classified as a CNS WHO grade 4 neoplasm, even though histologic signs of malignancy such as necrosis and microvascular proliferation are lacking (39, 45, 46). Roman numerals are no longer used to denote WHO grades; instead, Arabic numbers are used. It is stressed how crucial it is to find mutations other than the standard IDH1 R132H mutation in diffuse gliomas, particularly in patients under the age of 55. For example, noncanonical (such as non-R132H) IDH1 and IDH2 mutations should be examined in patients 55 years of age and younger with IDH-wildtype diffuse astrocytic gliomas. Other molecular markers, such as ATRX expression loss or TERT promoter mutations, presence of TP53 or histone H3 mutations, EGFR amplification, or CDKN2A/B changes, must be investigated (39, 45, 46).

The WHO classification of CNS malignancies in 2021 is a significant advancement with substantial implications for the management of patients with brain tumors. This new classification will improve diagnosis accuracy, provide better prognosis guidance, selection of more appropriate treatment, and allow enrollment of more homogeneous patient populations in clinical trials. This categorization will improve patient care and stimulate the development of more effective treatment regimens (39, 45).

## Cellular components of the tumor microenvironment

**Figure 1:**
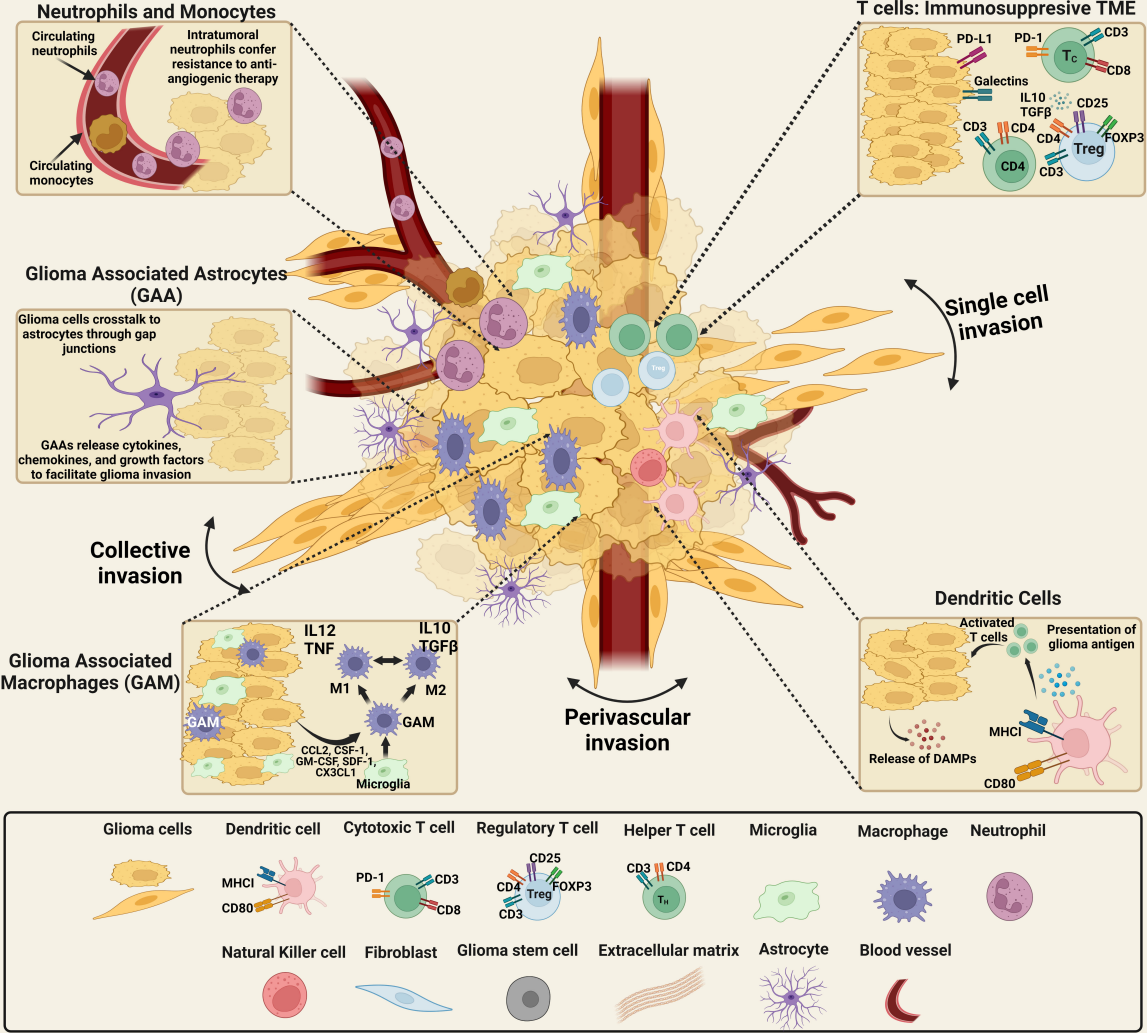
Schematics of the cellular components of the brain tumor microenvironmental landscape. The cellular components of gliomas: malignant and non-malignant cells, including tumor cells, a range of invasive peripheral immune cells, cells from the healthy brain including neurons and neuroglia, as well as pericytes and endothelial cells. The non-malignant cellular component consists of local immune cell types, including microglia and astrocytes, as well as lymphocytes, endothelial cells. High numbers of neutrophils in the systemic circulation indicates a positive therapeutic response. However, the presence of neutrophils in the glioma microenvironment confers resistance to anti-angiogenic therapy, suggesting high-grade glioma. Neutrophils promote the proliferation of GSCs with mesenchymal phenotypes and GSCs recruited through TGF-β secretion induce an immunosuppressive and, protumoral, M2 phenotype in macrophages. TGF-β and IL-10, immunosuppressive cytokines that impair T-cell response and antigen-presenting cell function, are overexpressed by glioma cells. The expression of MHC-I is downregulated in glioma cells while PD-L1 is upregulated, impairing T-cell response. Additionally, CTLA-4 expression reduces TCR activity. Recruitment and development of regulatory T cells are stimulated by TGF- and IL-10. Finally, the release of GM-CSF and CSF-1 by glioma cells promotes the recruitment of macrophages and polarization of M1 macrophages to an M2-like phenotype. The tumor–brain interface is distinguished by an invasive edge that harbors invasive glioma cells that migrate *via* white matter tracts or extracellular matrix fibers to infiltrate the brain parenchyma either collectively or as a single cell invasion. Glioma cells have been demonstrated to invade the perivascular space collectively, as a conduit for invasion. [Created with BioRender.com].

The tumoral composition of GBM is highly heterogeneous, both inter- and intratumorally as illustrated in **Figure 1**. This varied landscape, including cellular and non-cellular components, is termed the TME and encompasses the wide range of cell types and variations in ECM found within and near the tumor. The glioma TME includes both malignant and non-malignant cells - tumor cells, a variety of infiltrating peripheral immune cells, and the cells of the healthy brain such as neurons, neuroglia, and the additional components of the neurovascular unit (NVU), including pericytes and endothelial cells (47–50). Among the non-malignant cells are local immune cell types, such as microglia and astrocytes, as well as lymphocytes, endothelial, and other cells. Half of the tumor mass is composed of infiltrating cells, and most of the tumor-associated immune population are macrophages or microglia (2). However, the tumor environment is characterized as an immunosuppressive environment, inhibiting the immune response.

### Glioma cells

Glioma cells are thought to develop from altered glial progenitors, and there are several subtypes based on their differentiation state, including astrocytomas, oligodendrogliomas, and ependymomas (51, 52). Glioma cells can form tumors in any part of the brain and diffusely infiltrate surrounding parenchyma (53). Along pre-existing brain structures, glioma cells invade brain tissue through ECM components including myelinated fibers and brain vasculature (54). Glioma cells can eventually enter and occupy the subarachnoid region *via* the perivascular space (55, 56). Because gliomas tend to invade so much of the surrounding tissue, surgical resection is usually not enough to cure them. Instead, cells survive and grow back from the invasion zones beyond the resection margins (57). Glioma cells typically do not infiltrate the lumens of blood vessels, thus, systemic spread and metastasis in non-CNS organs are rare in brain neoplasia (58, 59).

### Glioma stem cells

GSCs are highly tumorigenic, invasive, and resistant to a variety of therapies (60). GSCs are frequently located in the “vascular niche” surrounding the tumor vasculature, which is known to offer microenvironmental signals to maintain GSC stemness, promote invasion, and enhance resistance to treatments. The SDF-1/CXCR4 axis transdifferentiate GSCs to endothelial cells and are triggered to become pericytes by TGF-β (61). Targeting pericytes derived from GSCs disrupts the blood-tumor barrier and enhances chemotherapeutic efficacy (61–66). Neutrophils promote the proliferation of GSCs with mesenchymal phenotypes (67) and GSCs recruited through TGF-β secretion induce an immunosuppressive and, protumoral M2 phenotype in macrophages (68, 69). WNT5A is associated with both differentiation of GSCs into endothelial cells (ECs) and recruitment of additional ECs (70). ECs recruit GSCs *via* the CXCL12 (SDF-1)-CXCR4 axis after which they differentiate into pericytes by TGF-β (61).

The cancer stem cell (CSC) hypothesis, which has been experimentally supported in the last two decades in connection to glioblastoma and numerous other cancer types, suggests that self-renewing CSCs originate and sustain tumor formation (71–77). CSCs can also arise from pediatric brain tumors (78). GSCs are capable of long-term proliferation, self-renewal, differentiation, and quiescence in G0 state, generating tumor spheres in culture due to their clonogenic capacity, and producing a tumor phenotypically similar to the original tumor when transplanted into recipient mice. Due to their propensity for self-renewal, and long-term replication, GSCs have been identified as the true “units of selection” during tumor growth (79). Recent research has demonstrated that different genetic and epigenetic variations of GSCs coexist in GBM and that they are responsible for cancer evolution (80). Experimental evidence suggests that GSCs serve as cellular drivers of the subclonal expansion in glioblastoma development and evolution. Hypoxia-inducible factor HIF2α and several HIF-regulated genes are more likely to be expressed in GSCs than in normal neural progenitors or non-stem tumor cells (63, 81–85). As GBM grows, the original network of vasculature is no longer sufficient to provide the growing mass, and hypoxia starts to occur in some regions (86, 87). Brognaro describes different phases of hypoxia, the first phase is pre-hypoxic, where the oxygen level is between 10-5%, HIF2α is a GSC marker during the second phase of GBM development, when the oxygen level is between 5-1%, while the third phase is very severe hypoxia, which lasts from 1% oxygen (when HIF1α is also turned on) to a very low level of oxygen tension (86, 87). This indicates that GSCs are the most sensitive cells to hypoxia and that they pass on the traits that make them fit to their offspring, which drives tumor evolution. In the same way, GSCs can quickly turn up the Glut3 transporter for glucose uptake when there isn’t enough glucose (88).

Singh et al. have shown that CD133^+^ cells may develop into tumor cells that phenotypically resembled the patient’s tumor (89, 90). CD133^+^ glioma stem cells promote radio resistance and glioma recurrence through regulation of the -DNA repair and checkpoint response network; Bao et al. have shown that targeting this DNA damage checkpoint response suppresses radio resistance *in vitro* and *in vivo* (91). The authors further showed that L1CAM is essential for maintaining the growth and survival of CD133^+^ glioma stem cells (92). Vascular endothelial growth factor (VEGF) is regularly secreted at considerably higher levels by stem like-glioma cell and was further stimulated by hypoxia (93). Jin et al. showed that proneural GSCs are more responsive to EZH2 suppression than mesenchymal GSCs are to BMI1 inhibition. Given that glioblastomas contain both proneural and mesenchymal GSCs, targeting both EZH2 and BMI1 was more efficacious than targeting either agent alone (94).

### Invasion and migration

Gliomas are distinguished by intertumoral heterogeneity and diffuse invasion of normal brain tissue. To achieve this, gliomas employ a variety of motility modes, including single-cell invasion, collective invasion, and perivascular invasion (**Figure 1**) (15, 95–97). It is not well understood if tumor growth and spread are random, or if glioma cells self-organize to help tumors grow and spread (98). Collective motion patterns have been seen both during normal development and in diseases like cancer, where the cells change from epithelial to mesenchymal phenotypes and then spread to other organs; gliomas have also been shown to exhibit organized, moving structures (15, 99–101). Recent studies using explants of spontaneous intestinal carcinoma found that the cells in the tumor core moved collectively. Using mouse glioma explants from genetically engineered mouse models, we recently studied the complicated behavior of glioma cells both in the center of the tumor and at the border with normal brain (15, 97). To get a more accurate picture of gliomas, we need a better understanding of their dynamic heterogeneity, which includes their histological features and their molecular makeup. Disrupting the collective dynamic patterns of gliomas with drugs may eventually stop the growth and spread of gliomas, leading to novel glioma treatment strategies.

The current treatment for glioblastoma is maximal surgical resection followed by chemo- and radiotherapy. The success of brain surgeries can be credited to American neurosurgeons Walter Dandy and Harvey Cushing. Dandy’s incomparable achievements set him apart from others’ recognition, including his epochal paper on ventriculography. Before this technique was developed, it was difficult to precisely locate brain tumors. After ventriculography was perfected, it was possible to locate virtually every tumor (102). Since Dandy’s discovery, neurosurgery for glioblastoma has made much progress in terms of technology. Today, glioblastoma surgery is guided by 5-ALA or intraoperative MRI and helped by a neuronavigation system and neurophysiological monitoring. Due to these improvements in technology, neurosurgeons can now remove about 90% of tumors without affecting the patients’ functional status. But even though surgery has improved, and patients undergo radiotherapy and chemotherapy, patient outlook remains very poor. Many tumors remain resistant to current treatments and grow back after a short period of regression, in both patients with visible or non-visible residual disease after surgery. Recent evidence of a branched evolution pattern leading to the multifaceted and variegated subclonal architecture of the primary bulk of glioblastoma has explained this resistance (103). Additionally, micro-infiltration of the brain parenchyma occurs in the very early stages of tumor growth. Recurrences happen mostly close by, and sometimes far away from the initial tumor site, even in patients whose primary glioblastoma bulk was completely removed by surgery (103).

To discover a cure for this terrible disease, new treatments must take into account both the fact that different active CSCs in the primary tumor or in tumor residues post-surgery can become resistant and brain tumor initiating CSCs are still present in the normal brain parenchyma, here they are sheltered by their dormant state, cellular and non-cellular interactions within the glioma-microenvironment, and the blood-brain barrier (103). Intratumoral heterogeneity and the clonal evolution of GBM determine patient-specific responses to treatment. In particular, after treatment, the remaining tumor population may not be a single clone that is resistant, but rather a group of different cancer cells with genetic changes that help them in the invasion of the normal brain parenchyma and resistance to treatments (104). This review mainly focuses on discussing the cellular and non-cellular interactions at the bulk tumor core and the infiltrating tumor border to define novel and efficacious treatment modalities against glioblastoma.

### Immune cells

In the normal brain, macrophages are a minor population but orchestrate the immune response under pathological conditions. Macrophages are either recruited to the brain as the tumor forms (105) or are recruited as monocytes and induced to become macrophages once in the TME (106). They can enter the brain due to GBM-induced disruption of the BBB (107). Glioma associated macrophages (GAMs) have traditionally been understood to exist between the M1 immune stimulatory and M2 immunosuppressive phenotypes (**Figure 1**) (108). We now know that this dichotomous approach to TAM classification is an oversimplification of multiple macrophage phenotypes, thanks to recent advances in single-cell technology and high-throughput immune profiling (109).

Microglia are CNS-resident immune cells that behave pro-angiogenically (110). Microglial responses to different neuropathologies have also been linked to disruption of the BBB (111). Neutrophils may confer resistance to anti-angiogenic therapy, possibly explaining why neutrophil infiltration correlates with glioma grade (67). This could also be explained by their stimulation of tumoral proliferation and invasion *via* neutrophil extracellular traps (NETs) (112). Ferroptosis of tumor cells caused by neutrophils leads to the formation of necrotic regions in tumors (113).

Due to disruption of the BBB, T cells can also infiltrate the brain and enter the TME (114) self-inducing a CD8+ immunosuppressive phenotype (115). Additionally, the immunosuppressive regulatory T cells are the most common kind of T cell in the TME and are responsible for preventing cytotoxic T cell proliferation (116). Although T cells and other immune cells should be one of the body’s primary defenses to attack tumor cells, in GBM, T cells often become inactivated near the tumor due to genetic reprogramming and immune cell exclusion.

Myeloid cells are the most common immune cells found in the glioma microenvironment, accounting for 60% of all infiltrating immune cells (117–119). Resident microglia, bone marrow-derived macrophages, myeloid-derived suppressor cells (MDSCs), dendritic cells, and neutrophils make up this population (12, 120). Despite their different developmental origins, microglia and macrophages share several phenotypic characteristics and can be distinguished by distinct cellular markers. Microglia make up 10% of the brain cell population and are derived from yolk sac erythro-myeloid progenitors during early embryonic development (121). Microglia are critical in maintaining brain homeostasis. However, under pathological conditions, they tend to polarize into two traditional categories: neurotoxic and neuroprotective, with changes in morphological features and marker expression (122–124).

Microglia and peripheral macrophages/monocytes activate, proliferate, and contribute to the disturbance of immune homeostasis under pathological conditions (9, 125, 126). Resident microglia, not macrophages, promote vascularization in brain tumors *via* the CXCL2-CXCR2 signaling pathway and are a source of alternative pro-angiogenic agents (110). Additionally, inhibiting the myeloid checkpoint of signal regulatory protein alpha (SIRPα) in microglia represses microglial stimulation by acting on the neuronal CD47 (127). SIRPα contains a receptor tyrosine-based inhibitory motif (ITIM) in its cytoplasmic region, which is phosphorylated upon contact with CD47, thereby increasing the binding and activation of SHP-1 and SHP-2, which limit phagocytosis by inhibiting myosin IIA deposition at the phagocytic synapse (128–130). Consequently, the observed upregulation of CD47 in GBM promotes the immunosuppressive properties of microglia in the tumor microenvironment (131, 132).

### Myeloid-derived suppressor cells

MDSCs are a diverse population of immature myeloid cells that release large amounts of immunosuppressive mediators and impair anti-tumor immunity. These cells can originate from either monocytic (M-MDSCs) or granulocytic (PMN-MDSCs) precursors (133). M-MDSCs have a better immunosuppressive capacity and are more prevalent in the blood of GBM patients, but PMN-MDSCs are more prevalent in the glioma microenvironment (134). Recent research by Bayik et al. demonstrates that chemotherapy can be utilized to target M-MDSCs in males, while IL1 pathway inhibitors can assist females by inhibiting PMN-MDSCs (135). MDSC subsets can contribute to the progression of primary tumors and promote metastatic dissemination, by inhibiting the antitumor immune response, boosting cancer stem cell (CSC)/epithelial-to-mesenchymal transition (EMT), and increasing angiogenesis (136).

Glioma-derived cytokines are the primary stimuli that induce the recruitment and proliferation of MDSCs, which can be MDSCs recruiters (including CCL2, CXCL8, SDF-1, and CCL2) and/or MDSCs expanders (including IL-6, PGE2, IL-10, VEGF, and GM-CSF) (134, 137). A cytokine screen further revealed that glioma stem cells released many molecules that enhanced MDSCs mediated immune suppression, including macrophage migration inhibitory factor (MIF), which acts to decrease immunological rejection by triggering and maintaining immune suppressive MDSCs in the GBM TME (138, 139). In glioma patients, elevated plasma levels of arginase and G-CSF may be associated with MDSC suppressor function and MDSC expansion, respectively (140). MDSCs inhibit cytotoxic responses mediated by natural killer cells and impede the activation of tumor reactive CD4^+^ T helper cells and cytotoxic CD8+ T cells (141). MDSCs along with glioma-associated macrophages have the ability to recruit Treg cells to the tumor site (141). Multiple mechanisms, including the development of oxidative stress, expression of T cell inhibitory ligands, inhibition of T cell migration, and depletion of essential T cell metabolites, contribute to this suppression (133, 134). In GBM patient tissue, elevated MDSCs levels in recurrent GBM predicted a poor prognosis. A CyTOF study of peripheral blood from newly diagnosed GBM patients demonstrated a concurrent decrease in MDSCs and rise in dendritic cells with time. Similar to the levels of individuals with low-grade glioma (LGG), GBM patients with prolonged survival exhibited less MDSCs. The identification of MDSCs as a prominent immunosuppressive population identifies them as a therapeutic target for glioma (139, 142). In the Phase I clinical trial (NCT02669173), the combination of low-dose, metronomic capecitabine and bevacizumab was well tolerated by GBM patients and was associated with a decrease in circulating MDSC levels and an increase in cytotoxic immune infiltration into the TME (143).

### Astrocytes

Astrocytes comprise the bulk of brain cells and play a significant role in GBM (144). Tumor-associated astrocytes (TAAs) promote GBM proliferation, survival, and invasion of brain parenchyma by enhancing the release of degradative enzymes, cytokines, chemokines, and growth factors (145). Additionally, brain tumor cells make direct contact with astrocytes through gap junctions, resulting in enhanced chemo- and radio-resistance (**Figure 1**) (146). Astrocytes also release exosomes that suppress PTEN *via* microRNA in GBM (147) through these gap junctions (148–150). Additionally, astrocytes interact with microglial cells, contributing to the immune-suppressing glioma TME (145). Recent findings indicate that astrocytes may be activated by brain tumor cells and play a crucial role in the development, aggressiveness, and angiogenesis of the tumor mass (151). Astrocytes transform into reactive astrocytes, which are defined by hypertrophy and upregulation of intermediate filaments [such as nestin, vimentin, and glial fibrillary acidic protein (GFAP)], and stimulation of cell proliferation (152). Active STAT3 in reactive astrocytes associated with worse survival after diagnosis of brain metastases in patients. Even at advanced phases of colonization, inhibiting STAT3 signaling in reactive astrocytes inhibits experimental brain metastasis from various primary tumor sites (153). Astrocytes secrete substances that retain the blood-brain barrier (BBB) tight junctions, which controls whether metastatic cells can invade the brain. Additionally, astrocytes also encourage the release of degradative enzymes, anti-inflammatory cytokines (including TGFβ, IL10, and G-CSF through activation of the JAK/STAT signaling pathway), chemokines, and growth factors, which eventually promote tumor cell growth, survival, and invasion (154–157). The balance of pro- and anti-inflammatory cytokines is shifted toward a pro-inflammatory milieu by inhibition of the JAK/STAT signaling pathway. Tumor-associated astrocytes contribute to anti-inflammatory responses is suggested by the intricate relationship between astrocytes and microglial cells, which can further contribute to the immunosuppressive glioma microenvironment (145).

### Fibroblasts

Fibroblasts secrete ECM components and recent multimodal investigations have begun to illuminate their presence in the meninges, choroid plexus, and perivascular spaces of the brain and spinal cord. It is still unknown where CNS fibroblasts come from and what they do, but it is obvious that they belong to a unique cell population, or populations, which have often been mistaken for other cell types due to the expression of overlapping cellular markers (20). Fibroblasts differ in transcriptional signatures across the layers of the meninges during development, with the pia mater fibroblast populations enriched in matrix metalloproteinases (MMPs) and a variety of ECM components including collagens and proteoglycans (**Figure 1**) (158). Subarachnoid-activated fibroblasts and perivascular inactivated fibroblasts surrounding the cortex-penetrating pia mater both express PDGFR-β (159). These perivascular Col1a1+ fibroblasts are largely absent in the brain at birth but are present later in development and are suggested to migrate along parenchymal vasculature (160).

### Blood-Brain Barrier

The blood brain barrier (BBB) is composed of endothelial cells, astrocytic processes, pericytes, and the basement membrane (161). Traditionally, the BBB was understood to facilitate the immune-privileged status of the brain; however, the extent to which the immune landscape of the brain is separate from the rest of the body has recently been called into question. The BBB can be compromised by GBM due to a number of factors including increased cerebral pressure, tumor cells displacing astrocytic feet as they proliferate in the perivascular space (162), and Semaphorin3A expressing extracellular vesicles released by tumor cells (163). Furthermore, the ECM of the BBB is altered in GBM including lessened expression of agrin and increased tenascin in the basal lamina (164). As GBM grows, new blood vessels are formed (neovascularization), which helps the tumor to expand. This neovascularization starts with endothelial cells, involves extracellular matrix changes, migration and proliferation of vascular cells forming capillaries (95, 165–167).

## Non-cellular components of the microenvironmental landscape in brain tumors: Molecular and biochemical aspects

### Extracellular matrix in brain tumors

**Figure 2:**
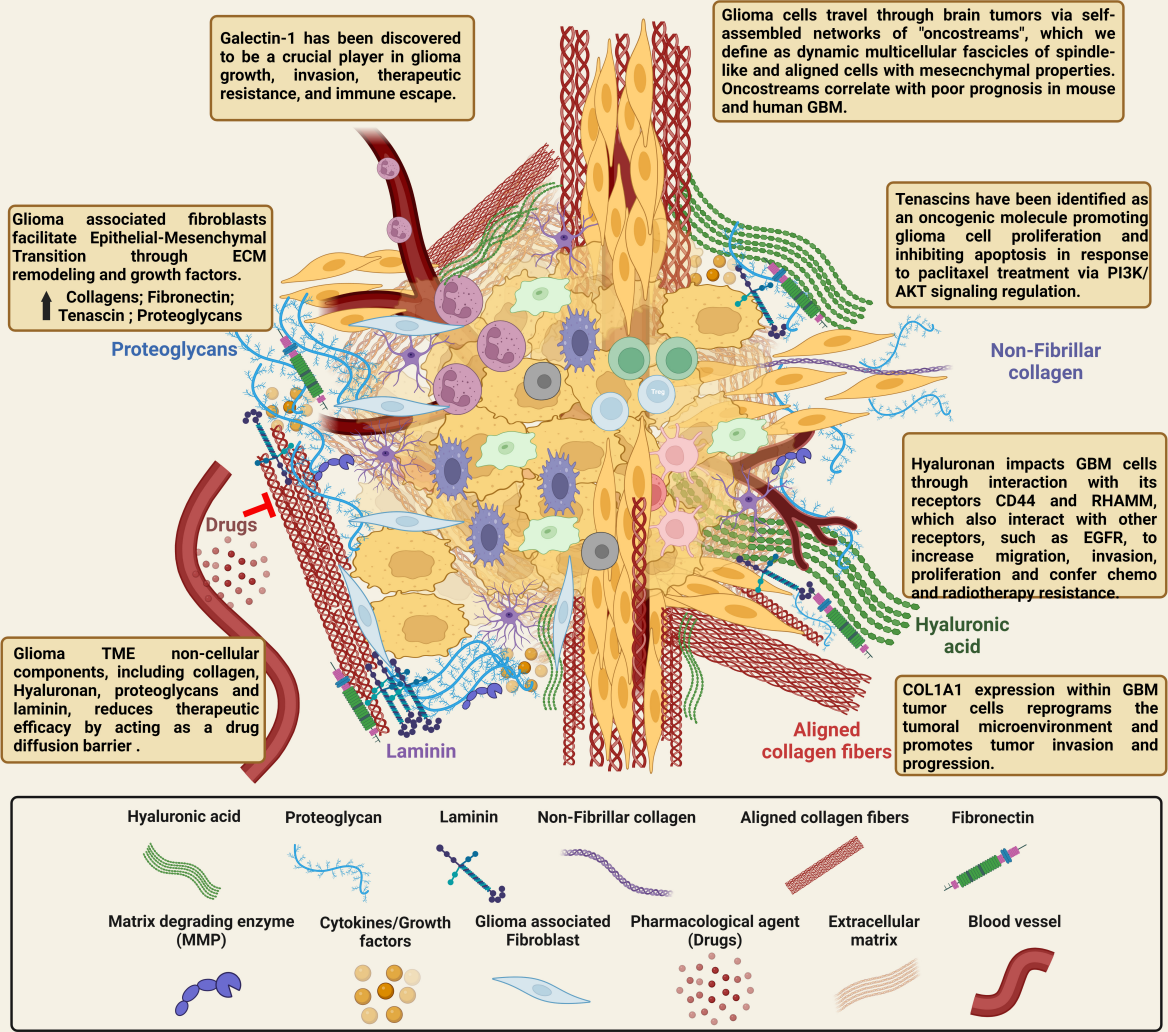
Illustration of the complex cellular and non-cellular ECM interactions in the glioma tumor microenvironment. The normal brain’s ECM differs from that of other organs because it contains very little of the fibrotic ECM proteins collagen, fibronectin, and laminin. On the other hand, the brain parenchyma is abundant in proteoglycans, connecting proteins, and glycosaminoglycans (hyaluronic acid). The ECM is transformed in GBM tumors compared to healthy brain tissue, and it is crucial for tumor invasion and migration. The ECM of GBM contains higher levels of collagen, fibronectin, laminin, hyaluronic acid, tenascin C, and vitronectin, all of which have been linked to glioma growth, invasion, and chemo- and radio-resistance. Due to the complex interactions between glioma cells and the non-cellular components in the GBM TME, targeting the ECM directly and attempting to alter the tumor production of ECM proteins, and thereby reduce tumor growth, should improve the infiltration of immune cells, drugs and provide improved therapies for highly malignant brain tumors. [Created with BioRender.com].

The non-cellular components of the tumor microenvironment are generally classified as the extracellular matrix, which functions as a biochemical and biophysical support of the cellular components of the TME as shown in **Figure 2**. The ECM includes interstitial fluid, minerals, fibrous proteins including fibers that provide tensile strength like collagen and elastin, and adhesive glycoproteins like fibronectin, laminin, and tenascin. The non-fibrillar ECM constituent includes the proteoglycans - heparan sulfate, chondroitin sulfate, and keratan sulfate - and the glycosaminoglycans including hyaluronic acid (168). Different organs have a particular composition of these elements that are directly related to their function. The general composition of the ECM arises from the balance between the cellular production/secretion and degradation. The ECM is highly dynamic and is stable under normal physiological conditions. However, in different pathological conditions such as cancer, the balance between production and degradation is lost. The mechanical forces generated by the ECM have recently been discovered to play critical roles in disease progression and malignant cell behavior (169–171).

The ECM of the normal brain is different from other organs as the brain has minimal levels of fibrotic ECM proteins like collagen, fibronectin, and laminin. These proteins are primarily found in the basal membrane around blood vessels and at the meninges. On the other hand, the brain parenchyma is rich in glycosaminoglycans (hyaluronic acid), proteoglycans and other linking proteins (6, 7). Compared to the healthy brain, the ECM is altered in GBM tumors and plays a crucial role in tumor invasion and migration (3, 4). Higher concentrations of collagen, fibronectin, laminin, hyaluronic acid, tenascin C, and vitronectin are present in the ECM of GBM and have been shown to have important roles in the onset and glioma progression (**Figure 2**) (4, 5). Additionally, several of these non-cellular components of the ECM have been especially linked to regulating tumor growth and are covered in more detail below.

GBM cells not only need to collect oncogenic genetic changes, but they also need permissive signals from the ECM around them in order to become more malignant (172). For instance, it has been shown that the ECM can change the amount of mRNA and the rates of protein synthesis and secretion. The ECM affects gene expression through transmembrane proteins (like integrins) and parts of the cytoskeleton. The cytoskeleton is connected to polyribosomes, which control mRNA stability and protein synthesis and to the nuclear matrix, which controls mRNA processing and rate of transcription. It has recently been shown in pancreatic cancer that disrupting these ECM cues alone can change oncogenic programs and slow down tumor growth (173). Similarly, identifying important ECM components in GBM can u er new therapeutic targets to halt tumor progression.

### Collagens and collagen receptors

Collagen is the most abundant of the ECM components and is classified in 29 different collagen subtypes. The molecular structure of collagen is a homotrimer or heterotrimer helical of 3 polypeptide chains which present a Gly-X-Y sequence (X and Y are typically proline or hydroxyproline). Collagens are classified in three main varieties: fibrillar, network forming, and fibril associated. Fibrillar collagen is mainly represented by collagen type I, in addition to collagens II, III, V and XI (174, 175). Collagen fibers are synthesized inside the cells as pro-collagen, which is then post-translationally modified in the rough endoplasmic reticulum *via* hydroxylation of proline and lysine and glycosylation. The fibers are then secreted from the Golgi to the extracellular space where they are cleaved and then crosslinked to other microfibrils and proteoglycans such as decorin and biglycan. This process forms large fibers that provide necessary tension for the tissue (5, 171, 176). Besides its function as a structural molecule, collagen plays a central role in cellular signaling and interactions with other ECM components. Specific domains of collagen bind cellular receptors including integrins, dimeric discoidin domain receptors DDR1 and DDR2, paired immunoglobulin receptor glycoprotein VI (GPVI), the leukocyte associated immunoglobulin like receptor 1 (LAIR1), urokinase-type plasminogen activator associated protein (Endo180). Different studies using mouse glioma models and The Cancer Genome Atlas (TCGA) data showed that fibrillar collagens such as type I are overexpressed in the glioma tumor mass compared to normal brain, and their levels positively correlate with tumor grade and malignancy (15, 177). Moreover, we recently demonstrated that inhibition of COL1A1 expression within the tumor cells reprogramed the tumoral microenvironment and inhibited tumor invasion and progression (15). Upregulation of certain growth factor such as TGF-β, can also contribute to ECM upregulation in gliomas (178).

Another subtype of collagens are the network-forming collagens, including collagen IV, VIII and X. These collagens are essential components of the basal lamina of the basement membrane and are characterized by the presence of NC (No-collagen) domain at the N and C terminal ends that enable the formation of collagen networks (5, 179). Collagen IV is localized at the base membrane surrounding the blood vessels and it is upregulated in gliomas (180, 181).The third subtype of collagens are the fibril-associated collagens (FACIT), including collagen XVI. These molecules present interrupted triple helices and, although they do not form fibrils by themselves, bind to other ECM proteins such as proteoglycans and small leucin rich proteoglycans (SLRPs) then are further stabilized by the lysyl oxidase enzymes (LOXs) (5, 182, 183). Collagen XVI has been reported to be overexpressed in glioblastomas compared to normal brain parenchyma, and its inhibition reduced glioma cell invasion (184, 185).

### Fibronectin

Fibronectin is less abundant in GBM tumors than collagen but does form an intricate fibrillar network connecting cells to the ECM (171, 186). Fibronectin is a dimer and multi-domain proteoglycan that is encoded by a single gene but has 20 isoforms *via* alternative splicing. The fibronectin fibrillar matrix is formed by binding to cell α5β1 integrins and other fibronectin molecules, collagen fibers, and heparan sulfates. The binding of fibronectin to other ECM components has been shown to be critical for the assembly of collagen fibers such as type I; collagen abundance also affects the assembly of fibronectin (187, 188). Studies have shown that the antibody targeting the collagen binding site on fibronectin suppressed the formation of collagen fibers (189). Other studies have shown that inhibition of collagen 1A1 decreased the levels of fibronectin expression and matrix assembly in a glioma mouse model (**Figure 2**) (15). In tumors, fibronectin is secreted by several cells including mesenchymal cells, fibroblast, endothelial cells, and the perivascular smooth muscle cells which, in turn affects the proliferation, migration, and cell-adhesion of tumor cells (190). Moreover, it has been shown in different cancers that the binding of fibronectin to cell integrins can affect cell signaling transduction and growth factors, such as the transforming growth factor beta (TGF-β), fibroblast growth factor (FGF), and platelet-derived growth factor (PDGF), which can all directly interact with fibronectin (190, 191). In gliomas, it has been shown by immunohistochemistry and using the PrognoScan data base that fibronectin expression correlates with tumor progression and prognosis as mediated by TGF-β signaling (192). Other research demonstrated by Cox regression analysis from the TCGA database that fibronectin was a risk factor for GBM, and its RNA levels were overexpressed in gliomas (193).

### Laminin

Laminin is the main component of the basement membrane along with collagen IV, fibronectin and perlecan. It participates in vascularization and wound healing and is upregulated in different cancer pathologies, regulating cell adhesion, differentiation, and migration (**Figure 2**) (171, 194). Laminin is a glycoprotein formed by three different chains, the α, β, and γ chains, each encoded by different genes. The α chains has five forms (LAMA1-5) and, the β and γ chains each have three forms (LAMB1-3 and LAMC1-3, respectively) which are found in laminins in different combinations (195).

It has been reported that during glioma progression, laminin-9 (α4β2γ1) is changed to laminin-8 (α4β1γ1), which is highly upregulated. Laminins 8 and 9 are differentiated by the β chain. Glioma tumors with higher expression of Laminin 8 show accelerated cellular spread and tumor recurrence (196). Other studies found that the α4 subunit of laminin (LAMA4) is overexpressed in the cerebral spinal fluid from GBM patients compared to patients without brain tumors and the expression levels correlated with the overall GBM tumor volume (197). A study of glioma cell migration found that laminins play an important role in this process. The authors indicated that GBM tumors express α2, α3, α4 and α5 laminins chains, and they demonstrated that α3 (Lm332/laminin-5) and α5 (Lm511/laminin-10) laminins highly enhanced glioma cell migration mediated by integrin binding (198).

### Tenascin

The tenascin family comprises a large group of glycoproteins generated by alternative splicing resulting in different variants, including TN-C, TN-R, TN-W, TN-X, TN-Y. Tenascin is highly expressed during embryonic development, wound healing, and cancer (199–202). Different member of the family display different and even opposite functions and their expression level are regulated by growth factors, cytokines and, other ECM components (**Figure 2**) (202, 203). Most tumors express high levels of this ECM glycoprotein and gliomas in particular have an enrichment of Tenascin-C (TN-C), which participates in tumor progression and correlates with tumor malignancy (204, 205). Tenascin binds to cell integrins, modifying cellular function, and also binds to other ECM molecules, such as brevican or neurocan, modifying cell migration and, focal adhesion (1). Moreover, tenascins have been identified as an oncogenic molecule promoting glioma cell proliferation and inhibiting apoptosis in response to paclitaxel treatment *via* PI3K/AKT signaling regulation (206).

### Hyaluronic acid, glycosaminoglycans, and proteoglycans

Other non-fibrillar ECM components found in the normal brain and in glioma tumors are the glycosaminoglycans (GAGs), proteoglycans, and hyaluronic acid (**Figure 2**). GAGs are linear polysaccharides, with each block comprised of disaccharides (GlcNAc or GalNAc) and uronic acids (GlcA and IdoA). Proteoglycans are abundant in the ECM and are formed by a core protein bound to one or more attached GAGs (207). Proteoglycans play a key role in the brain as growth factor reservoirs and stabilizers for ligand-mediated signaling by acting as co-receptors (208). In turn, they regulate normal cell signaling and migration and promote receptor tyrosine kinase (RTK) signaling and progression in glioma (208). Hyaluronic acid is another essential component of the brain ECM and is the simplest glycosaminoglycan. Hyaluronic acid is formed by a negatively charged long polymer chains forming random coils intertwined in solution. The many hydroxyl groups on the chains retain high amount of water increasing the elastoviscosity and maintaining the osmotic balance of the tissue (190, 209). Its production is upregulated in glioma tissue (210), inducing signals from the ECM to the cytoplasm and enhancing tumor cell migration and proliferation (211). High hyaluronic acid levels have been shown to correlate with poor prognosis in GBM patients (212). Molecularly, hyaluronic acid binds to different membrane receptors including the glycoprotein receptor CD44 and the receptor for hyaluronan-mediated motility (RHAMM), which has a central role in glioma cell motility, invasion, and inflammation (19, 213). It has been demonstrated that high levels of CD44 are necessary to generate infiltrative glioma mouse models and that treatment with anti-CD44 antibodies inhibited tumor progression, possibly due to altered hyaluronic acid binding (214).

### Biochemical interactions between glioma cells and the ECM mediated by receptor-ligand binding

Extracellular RTKs such as EGFR, PDGFR, and IGF-1R are cell surface receptors for growth factors, hormones, cytokines, and other signaling molecules. When a ligand binds, RTKs are dimerized and phosphorylated, in turn, activating the downstream signaling pathways Ras/MAPK/ERK and Ras/PI3K/AKT (215). This upregulates cell migration and proliferation, cell growth and survival, translation and differentiation, and angiogenesis while inhibiting p53 and PTEN activation and apoptosis. EGFR is amplified and mutated in 45-57% of GBM cases, inducing proliferation and resistance. However, EGFRvIII – a truncated mutant variant III – is correlated to increased patient survival, possibly due to triggering an immune response (215–217). Platelet-derived growth factor receptor alpha (PDGFRα) is amplified in 10-13% of GBM cases in the TCGA database and stimulates a malignant GBP autocrine loop. Similarly, the activation of insulin-like growth factor 1 receptor (IGF-IR) through interactions with IGF-1 promotes the recruitment of IRS-1, activating AKT and ERK, resulting in increased GBM cell growth, proliferation, and migration (218).

Silver et al. investigated the components driving, and inhibiting, diffuse glioma invasion, and found that glycosylated chondroitin sulfate proteoglycans (CSPGs) are heavily present in noninvasive lesions compared to highly infiltrative tumors (219–221). They found CSPGs act as molecular barriers, organizing the brain tumor microenvironment, inducing activation of tumor-associated microglia and reactive astrocytes exit from the tumor (220, 222). Specifically, leukocyte common antigen-related (LAR) receptors bind to glycosylated CSPGs, anchoring CSPGs to resist passive diffusion, inhibiting tumor cell infiltration. It’s also speculated that LAR functions as an adhesive molecule, tightly binding to noninvasive tumor cells to the CS-GAG-rich ECM (219, 220). The receptor protein tyrosine phosphatase μ (PTPμ) is another cell adhesion molecule and is downregulated in GBM due to proteolytic cleavage. It has been shown that downregulation of these catalytically active PTPμ proteolytic fragments degreases GBM migration and survival, representing a possible therapeutic target (223).

## Interstitial fluid and soluble factors

Numerous soluble substances extravasated from intravascular compartments or released by tumor or stromal cells within the TME create a dynamic interstitial fluid compartment of cells and ECM components (224). The cellular makeup of most tumor regions is continually changing due to these variations in soluble component concentrations, which results in dynamic changes in chemotactic gradients. The ECM is a reservoir for cytokines that can regulate the migration and function of immune cells, primarily those with immunosuppressive effects such as interleukin-10 and TGF-β (225, 226). TGF-β has been shown to positively correlate with collagen-binding integrin α2β1 levels and may play a role in activation of collagen and fibronectin synthesis (5, 227). High TGF-β activity confers poor prognosis in patients (228). Clinical trials testing TGF-β inhibitors in glioma have not yet shown significant efficacy (229, 230). However, many aspects of its role in tumor progression are not fully understood, including its presence in ligand-shielding exosomes (231), and TGF-β remains a promising target for future research.

Tumor-associated macrophages are a primary source of anti-inflammatory molecules such as TGF-β, ARG1, and IL-10, as well as pro-inflammatory molecules such as tumor necrosis factor-α (TNF-α), IL1-β and CXCL10. In addition, TAMs also produce remodeling and angiogenesis molecules that give tissue support such as vascular endothelial growth factor (VEGF), MMP2, MMP9 and Membrane Type-1 MMP.

The GBM microenvironment is also characterized by tissue hypoxia due to the irregular vascularization and elevated tumoral oxygen consumption. Tissue hypoxia activates the STAT3 pathway, HIF-1α synthesis, activation of Tregs and production of VEGF, ultimately inhibits recruitment of dendritic cells (232). In CD4^+^ T-cells, HIF-1α promotes IL-17 and Th17. Hypoxic microenvironments are a common feature of GBM, which is caused by morphologically and functionally inappropriate neovascularization, irregular blood flow, and proliferating glioma cells that require much oxygen (233, 234). Hypoxia inducible factor (HIF)-1α is produced during tumor hypoxia by activating the immunosuppressive pSTAT3 pathway (235). HIF1α is implicated in the regulation of T-cell immune checkpoints, and promotes PD-L1 expression in cancer cells, macrophages, dendritic cells, and MDSCs (236–238). The CTLA-4 receptor, which is increased on CD8+ T-cells under hypoxia perhaps *via* HIF1, is another checkpoint regulated by hypoxia (HIF1) (232, 239). Teffector cells are inhibited and Treg cells are activated when CTLA-4 on T-cells binds to the ligands CD80 and CD86 on the surface of APCs (240). Clinical trials using CTLA-4 and PD-1/PD-L1-targeted treatments for several cancer types demonstrated encouraging results (241). A lot of immune-suppressing cells, like Tregs, MDSCs, and TAM, invade the GBM microenvironment and upregulate multiple immune checkpoints, like PD-1, Tim-3, CTLA-4, and IDO-1, and immune-suppressing ligands, like PD-L1 on GBM cells and tumor-infiltrating myeloid cells, which conceal GBM tumor antigens. This contributes immunosuppressive features to the GBM and suppress the T cells to proliferate and invade the TME (242). In macrophages, HIF-1α triggers the release of inflammatory cytokines augmenting myeloid cell recruitment (243). Hypoxic TAMs produce MMP9 when induced by HIF-1α. MMP9 increases bioavailability of VEGF, resulting in neovascularization (244). Therefore, the success of GBM immunotherapy also depends on lowering or eliminating the infiltration of immunosuppressive TME and raising the quantity and activity of effector T cells (245).

Galectin-1 (Gal-1) has also been discovered to be a crucial player in the development, invasion, and therapeutic resistance (246), as well as the escape and inhibition of the immune system (**Figure 2**) (247, 248). We recently demonstrated the existence of an innate anti-glioma NK-mediated circuit initiated by glioma-released microRNA (miR-1983) within exosomes and which is under the regulation of Gal-1 (249). We showed that miR-1983 is an endogenous Toll-Like Receptor 7 (TLR7) ligand that activates TLR7 in plasmacytoid and conventional Dendritic cells (pDCs and cDCs) through a 5′-UGUUU-3′ motif at its 3′ end. TLR7 activation and downstream signaling through MyD88-IRF5/IRF7 stimulate secretion of interferon (IFN-β). IFN-β then stimulates NK cells resulting in the eradication of gliomas (249).

Another important aspect of the ECM is the unique distribution of oncometabolites that are produced in the TME. A large subset of GBM display mutations in isocitrate dehydrogenase isoforms (IDH) 1 and 2, resulting in a buildup of the D enantiomer of 2-hydroxygluterate (D-2HG) (35, 37). IDH1-R132H suppresses tumor growth in gliomas *via* epigenetically activating the DNA damage response (36). Granulocyte-colony stimulating factor (G-CSF) reprograms bone marrow granulopoiesis, resulting in non-inhibitory myeloid cells within mIDH1 glioma TME and enhancing the efficacy of immune-stimulatory gene therapy (TK/Flt3L) (250, 251). Increased D-2HG levels exert a tumorigenic effect by supporting hypermethylation of tumor suppressor genes such as Tet methylcytosine dioxygenases (252). D-2HG may also affect proper collagen maturation, leading to an increase in tumor progression (253). Inhibition of D-2HG has been found to increase median survival in mouse models of GBM (254).

## Exploring the physical effects and multi-dimensionality of the tumor ECM

### Impact of ECM on cellular mechanics in glioblastoma: Biophysical interactions

Glioma cells and the ECM components of the brain biophysically interact with each other - pushing, pulling, degrading, and secreting – strongly influencing each other’s form and function. It has been recognized that physical qualities of the ECM including stiffness, viscoelasticity, mechanical plasticity, and nonlinear elasticity affect cellular processes like proliferation, apoptosis, migration, and spreading (255). Mechanistically, cell surface integrins recognize and bind to ECM proteins, clustering together to form focal complexes which grow and mature into focal adhesions (FAs). Cells sense the biochemical and physical characteristics of the ECM based on the extent of focal clusters, related signaling, and downstream transcription factor activation (255, 256) Pulling forces are often exerted by actomyosin-based contractility and pushing forces through actin polymerization and microtubules, and cells use these interactions to spread and migrate through the tissue (257–260).

Extracellular matrix fibers are not purely elastic, exhibiting viscoelasticity and irreversible plastic deformations (255, 261, 262). Viscoelasticity is a time-dependent mechanical property - tissues and the ECM may recover their shape when stretched slowly but can also experience strain-stiffening and permanent damage when rapidly extended. Shenoy et al. detail the various mechanical tests and constitutive equations used to characterize tissue and ECM deformations in their recent review (255) The brain is one of the softest viscoelastic tissues in the human body with a mean normal brain stiffness of 7.3 kPa and increases to between 11 - 33 kPa for different grade brain tumors as measured by intraoperative shear wave elastography (263). Because of this low relative stiffness, it exhibits high levels of dissipative energy, allowing glioma cells to migrate and reorganize ECM fibers within the soft tissue. While many *in-vitro* studies are performed in 2D environments, it’s essential to consider that glioma cells do not exist in a flat environment and 3D and *ex-vivo* studies can create more realistic models of GBM (264). Additionally, in a 2018 study, Ma et al. discovered that GBM cells cultured in 2D and 3D environments had drastically different cellular morphologies, genetic expression, and protein secretion (265). The authors point out the lack of the ECM and 3D interactions in the 2D culture as a main contributor to these inactivated signaling pathways and differing cellular morphologies showing their importance *in vivo* and for future studies.

#### ECM-glioma interactions on 2D substrates

The probing of substrate stiffness’ effect on cellular biomechanics started with studies of cells migrating on polyacrylamide hydrogels (PAM gels) coated with ECM proteins or functionalized for cell adhesion with arginylglycylaspartic acid peptide (RGD) (266, 267). These gels have tunable elastic moduli which can be modulated based on the environment of interest. In Umesh et al.’s 2014 study, they culture human GBM cells on fabricated polyacrylamide substrates ranging from 0.08 kPa–119 kPa (266). They found that increasing substrate stiffness increases cellular proliferation and regulates cell cycle progression by altering EGFR-dependent signaling. O’Neill et al. showed that four out of five of their primary GBM cell lines had rounded morphology on the softest PAM gels (0.2 kPa) and then spread out on stiffer gels (1.0 and 8.0 kPa) (267). Cell speed was also regulated by substrate stiffness in four of the cell lines with cells migrating faster on the stiffer substrate, as similarly described in many other *in-vitro* studies (268–270). While 2D studies have been shown to inaccurately portray GBM dynamics *in-vivo*, some methods to study physical forces and mechanics are not yet possible in 3D or *in-vivo*. Traction force microscopy (TFM) is a key method for understanding the complex array of forces at play when GBM cells migrate through the TME (271, 272). Cells are seeded on a hydrogel substrate embedded with fluorescent beads which are pushed and pulled by the cells as they migrate across them. Forces exerted by the cells are measured based on the displacement of the beads, the time-period, and the density of the hydrogel. These forces may be similar to those exerted by cells on the ECM as they migrate, and more in-depth studies are necessary to measure the exact forces on ECM fibers.

#### ECM-glioma interactions in 3D environments

However, although more complicated to perform, 3D studies retain more characteristics of a GBM tumor *in-vivo* and garner more accurate results (273–275). Much research into GBM-ECM interactions has turned to 3D *in-vitro* environments, ex-vivo studies, and even *in-vivo* mouse studies. The ECM mainly acts as a guiding scaffold or a barrier in which GBM cells migrate. Thus, many groups create 3D bioengineered scaffolds made of ECM proteins to model the TME (276–280). These scaffolds are usually manufactured by crosslinking ECM proteins inside polyethylene glycol (PEG), collagen, Matrigel or other gels and are then filled with either individual cells or cell spheroids. In 2013, Florczyk et al. designed porous chitosan-hyaluronic acid scaffolds which mimic the GBM tumor environment (276). These complex 3D scaffold cultures promoted cellular malignancy and drug resistance in a way that better models GBM cells *in-situ*. They also found that the physical interactions between the ECM and cells promoted upregulation of stem-like genetic properties and increased invasiveness and tumor spheroid formation. This study and others evaluated the effect of drugs and radiation on the cultures, including chemotherapies such as temozolomide, bevacizumab, and erlotinib (277). While erlotinib was shown to enhance radiosensitivity in 2D, 3D cultures were not affected, illustrating a key need for 3D studies to be performed before drug consideration for clinical trials.

A study by Ananthanarayanan et al. also developed HA scaffolds which could be altered to independently control biochemical and mechanical properties (273). This group studied migration in both 2D and 3D HA scaffold cultures, highlighting a key difference. In 2D, glioma cells exhibited a mesenchymal phenotype and migrated *via* lamellipodia at the leading edge. However, in 3D, the cells migrated with a “sling-shot” type migration in which the cells protruded, retracted, branched out, and then suddenly moved forward through the gel, in a process very similar to that observed in some *ex-vivo* glioma studies (281–283). This study also found that when HA density increased above 5% (corresponding to 5 kPa), glioma cell invasion was abolished, concluding that cell migration is sterically hindered at high concentrations of ECM proteins. However, this study emphasized that their HA hydrogels were non-fibrillar, claiming that this matched the native structure of the brain, but new research has shown that many higher-grade glioblastoma tumors contain fibrillar collagen and fibronectin components, as discussed above (14, 177, 284, 285). Another 3D method of probing ECM interactions with cells consists of creating 3D gels filled with electro spun fibers, mimicking collagen or fibronectin (286–288). Unlike 3D experiments using solely gels, adding in electro spun fibers allows researchers to study the effects of fiber orientation, size, and density. Some fibers have been shown to enhance axon growth and elongation, which could trigger tumor recurrence, while others were designed to mimic white matter tracts and enhanced GBM invasion through the tumor (287, 288). More studies on the interactions between glioma cells and synthetic fiber tracts or embedded fibers, and how these affect migration and invasion will be interesting.

#### *Ex-vivo* brain slices accurately mimic *in-vivo* environment

The aforementioned 3D platforms are a more promising tool for GBM drug development than 2D cultures and are faster and more efficient than most *in-vivo* studies. 3D studies also allow for the isolation of different ECM components to individually probe their importance in GBM development. However, they still lack the complex, multi-faceted characteristics of a tumor, and recent research has focused on *ex-vivo* or *in-vivo* methods to study ECM-GBM interactions. These studies use the native tumor microenvironment in order to retain the precise properties of the ECM and bulk tissue that cells experience. Comba et al. recently utilized 3D-organotypic slice cultures, and time-lapse confocal imaging and mathematical models to spatiotemporally quantify glioma cell organization and dynamics (15). While the precise physical qualities of the ECM were not measured in this study, retaining the native GBM environment preserves *in-vivo* cellular qualities and dynamics. We show that collagen presence and density is essential for the formation of malignant cellular structures called oncostreams which promote GBM migration and invasion. We further demonstrate that targeting the production of Collagen 1 by these tumors reduces malignant features of these tumors and improves survival. More recently it has also been shown that breast cancer cells produce an isoform of Collagen 1 that also has pro-tumoral effects. These organotypic studies have an advantage over the *in-vivo* studies discussed later; since they are not within an animal or human patient, mechanical characterization tests, such as atomic force microscopy (AFM) or nanoindentation, are easily conducted. In another study, Sin et al. designed a scaffold-free 3D culture system to model the invasion of glioma cells across a simulated tumor-stromal interface, taking into account the properties of the host tissue (289). By culturing spheroids of glioma cells (representing the tumor) next to neural progenitor cell organoids (representing the normal brain) and imaging with time-lapse confocal microscopy, they captured the 3D invasion of glioma cells into mouse neural spheroids and corroborated this with spheroid cryo-sectioning. Spheroids are easily created using many cell lines and these can also be embedded in different ECM-containing gels to study migration and invasion, thus representing a viable and effective tool for future GBM research.

#### Probing ECM-GBM cell interactions *in-vivo*

Measuring the mechanical properties of cells and the tumor microenvironment in live animals or patients is a formidable task, but several groups have devised techniques to understand GBM-ECM interactions *in-vivo* (15, 290–293). We recently demonstrated the intravital multiphoton imaging to study glioma cell dynamics deep into the brain with high-resolution and low photobleaching (15). Joyce et al. also used two-photon microscopy as well as magnetic resonance imaging (MRI) to longitudinally image glioblastoma tumor initiation and development (293). Using both methods, the authors could measure overall tumor growth or regression with different therapeutic measures and also probe the TME at a single-cell level. Blood vessels were visualized with fluorescently labeled dextran and meningeal collagen was imaged with second harmonic generation (SHG) imaging. SHG was first used in a biological setting in 1986 and is highly specific at visualizing fibrillar collagen (294). Briefly, SHG is a second-harmonic generation nonlinear scattering process which inherently recognizes the non-centrosymmetric qualities of fibrillar collagen without the need for fluorescent markers. Due to the importance of fibrillar collagen in GBM, an important next step for the field is to utilize combined longitudinal multiphoton and SHG imaging to understand collagen composition and dynamics inside the tumor.

Another technique called magnetic resonance elastography (MRE) has been used to non-invasively measure tumor and normal brain stiffness in human patients. MRE is a developing technique which sends a single-shot spin-echo echo-planar-imaging pulse sequence to induce shear waves through the brain (292, 295, 296). The properties of the shear waves are then correlated to the elastic modulus of the material through which they are traveling. Huston et al. conducted MRE on 14 patients with meningioma tumors and measured intratumoral stiffness with 3-mm isotropic resolution. While a cellular resolution of stiffness measurements has not yet been obtained *via* MRE, the authors did observe that the tumor was significantly stiffer than the surrounding normal brain. Additionally, MRE can measure stiffness difference by up to 5 orders of magnitude between tissues, whereas MRI and ultrasonography can only measure around 2 orders of magnitude of difference. The stiffness of the material in which glioma cells migrate has been shown to influence their speed, persistence, and morphology in 2D and 3D, and future studies *in-vivo* could confirm or refute these previous findings.

## Impact of ECM on tissue remodeling and immune suppression

The extracellular matrix functions as both a physical and biological barrier for the immune system (**Figure 2**). The immune system function is dependent on receptor-ligand interactions, which are influenced by cellular motion, bulk fluid flow and the local stiffness of the ECM. The physics of the tumor stroma is regulated by collagen fiber cross-linking, which is catalyzed by lysyl oxidase (LOX). The balance between matrix degradation and production is regulated by the antagonistic actions of matrix metalloproteinases and tissue inhibitors of MMPs (TIMP) (297). ECM remodeling affects immune cell trafficking and can impede immunological synapses (298). Lymphocyte motility and infiltration is determined by their interactions with the ECM. Tumor-infiltrating T cells are less abundant in areas with densely packed ECM fibers (299). Collagen I degradation by MMP8 or MMP9 generates the acetylated Pro-Gly-Pro (proline-glycine-proline) tripeptide, which shares structural homology with CXCL8 and is a major chemoattractant for neutrophils (300). Peptides generated from elastin digestion by MMP12 can also function as chemoattractant (301), and MMP2 stimulates monocyte derived dendritic cells (DC) *via* toll-like receptor 2 (TLR2) (302, 303).

However, ECM presence can also assist in immune cell infiltration as natural killers (NK), DCs and T cells migrate along fibrillar collagen (304, 305). T cells accumulate in loose fibronectin and collagen regions (299, 306). Targeting collagen fibers through LOXL2 increases T-cell infiltration (307). It has been shown that in a muscle wound, collagen scaffolds skewed the ratio of CD4:CD8 T cells toward a higher fraction of CD4^+^helper T cells. Furthermore, the T cells in the collagen-implanted wounds expressed higher levels of anti-inflammatory cytokines (308). Collagen density also influences macrophage infiltration. Low-collagen genetically engineered mouse models of glioma exhibit lower macrophage infiltration (CD68+ cells) (15). Neutrophils and monocytes have been shown to preferentially migrate through areas with low collagen IV (309, 310).

Most immune cells express receptors that interact with collagen. Discoid in domain receptors (DDR) are highly responsive to collagen and profoundly involved in cell migration (311–313). Blocking of these receptors has been shown to impair migration of neutrophils, monocytes, and T cells in a collagen matrix (311, 312, 314, 315). Leukocyte-associated Ig-like receptor-1 (LAIR-1) is expressed by the majority of PBMC and thymocytes (316, 317). LAIR-1 is also expressed by CD4^+^ and CD8^+^ T cells, with the highest expression in naïve T cells (318, 319). Furthermore, crosslinking of LAIR-1 on primary T cells results in an inhibition of T cell function (319). Interaction of LAIR-1-expressing NK cells with collagen inhibits NK cytotoxic potential (320). In monocytes, LAIR-1 ligation with an agonistic antibody inhibited TLR-mediated activation (321). Additionally, M1 macrophages on surfaces coated with LAIR-1 ligand peptide decreased secretion of TNFα and T cell attracting chemokines (322). Integrins also play an important role; several have been found to promote ECM adhesion in T cells in response to TCR stimulation (323). Macrophages also express various integrins, most commonly members of the β2-integrin family (324). The α2β1-integrin has been shown to mediate the migration and mechanosensing of macrophages cultured on 3D collagen matrices (325). It was also shown to be involved in M2-polarization in 3D culture in a gelatin-based hydrogel (326). Monocytes, macrophages, and DC also express osteoclast-associated receptor (OSCAR), which is another collagen binding receptor (327, 328). In contrast to LAIR-1, OSCAR-signaling is mainly associated with immune activation (329). OSCAR on monocytes and neutrophils is involved in the induction of the primary proinflammatory cascade and the initiation of downstream immune responses (330).

Other components of the matrix also interact with immune cells. Tenascin-C synthesis is known to be up-regulated in glioma (331) and T-cells accumulate on the border of tumor and normal brain in association with high TNC deposition (332). Additionally, glycosaminoglycans, hyaluronan, and versican have been shown to be immunosuppressive (297).

## Therapeutic opportunities targeting the extracellular matrix in the tumor microenvironment

### Enhanced targeting specificity *via* matrix-specific ligands

The extracellular matrix is a target for glioblastoma treatment for many reasons, and many ECM components are uniquely upregulated compared to healthy tissue and ubiquitously expressed across the tumor stroma. This presents the opportunity for the development of specific ligand-coupled treatment without cross-reactivity with normal brain parenchyma (14, 333, 334). Extra-domain B of fibronectin (FN-EDB) is almost exclusively expressed in areas of angiogenesis. Antibody L19-SIP is a notable molecule that was developed to specifically bind to FN-EDB (335, 336). It was shown to significantly slow tumor growth and increase survival in orthotopic murine models of glioma when fused to a variety of cytokines, including IL2 (337). The efficacy of L19 as a treatment guide is being investigated in multiple clinical trials, with promising results in other cancer types (338). A phase I/II trial investigating the safety and antitumor activity of L19-TNF on IDH-wildtype WHO grade III/IV glioma is currently ongoing (NCT03779230). Numerous other methods have been developed to target FN-EDB, including selective aptamer-like peptides encasing siRNA, which were found to significantly slow tumor growth in a GBM xenograft mouse model (338, 339).

Tenascins are normally involved in embryonic development but are upregulated in adult brains in malignant tissue. Numerous ligands have been developed to selectively bind to TN-C, including antibody 81C6, which has shown promising results as a treatment guide in phase I and II clinical trials (340, 341). Peptide PL3 has recently been found to significantly slow tumor growth and improve survival of xenograft glioblastoma-bearing mice when used to guide pro-apoptotic nanoworms (342).

Brevican is a chondroitin surface glycoprotein abundantly expressed in adult CNS. However, the isoform dg-Bcan is unique to high-grade gliomas (343). BTP-7 is a novel peptide that binds to dg-Bcan, and it has shown high affinity and accumulation in murine GBM xenografts (344). Although its specific role in GBM is unclear, brevican levels were also found to be upregulated in more aggressive patient tumor samples when analyzed by immunohistochemistry (IHC) (345). In addition to acting as a treatment target, strategies modulating brevican expression may be useful once its effects are better understood.

### Modulation of specific ECM components as GBM therapy

Along with its specificity, many components of the tumor ECM have been shown to promote tumor growth and infiltration, presenting the potential for targeted treatments. While much of the specifics behind these effects are largely unknown, some possible mechanisms have been suggested. Many ECM components are over-expressed in tumor tissue, encapsulating the tumor with a physical barrier. This barrier limits access to nutrients and oxygen, leading to hypoxic conditions which promote tumor aggressiveness and invasion. Homogenous treatment distribution is also hindered by this barrier (346). Finally, the unique properties of the tumor ECM aid growth and migration into healthy tissue (347). These tumor-shielding and promoting effects also suggest that inhibition of ECM production and induction of degradation may be useful tools for GBM treatment.

Degradation of HA may be a useful treatment to allow better therapy and immune penetration to the tumor center. A study of orthotopic GBM models found that treatment with hyaluronidase-expressing oncolytic adenovirus (ICOVIR17) displayed improved survival and infiltration of CD8+ T cells and macrophages when compared to a control virus (348). While no clinical trials have studied HA degradation in glioma, a phase II trial found significant improvement in progression-free survival in pancreatic cancer patients treated with hyaluronidase (PEGPH20) in combination with paclitaxel/gemcitabine (17). However, a follow-up phase III trial was terminated due to negative study outcome (18). HA synthesis inhibitor 4-Methylumbelliferone has also been proposed as a potential drug of interest. It was found to reduce proliferation and migration in GBM cell line GL26, as well as amplify cytotoxicity when paired with TMZ (211) and more research and drug development may lead to successful clinical trials.

Given their abundant interactions with hyaluronic acid, hyaluronic acid receptors are another potential treatment target. High levels of CD44 (a transmembrane hyaluronic acid receptor) were associated with a significant decrease in median survival in tissue specimens from 62 GBM patients (349), and CD44 inhibition reduced tumor growth in a mouse model of GBM (350). Additionally, a proposed mechanism of GBM migration involves attachment of cell “micro-tentacles” to hyaluronic acid *via* CD44 (351). While no clinical trials have been performed on glioma patients, the CD44 inhibitor RG7356 induced modest clinical benefits in a trial of 65 patients with advanced CD44-expressing solid tumors (352). Along with CD44 levels, hyaluronan-mediated motility receptor (HMMR) levels were upregulated in the tumors of patients with shorter overall median survival when analyzed by IHC (345), and maybe another significant target (353).

As mentioned, despite its ubiquity in most of the body, collagen expression is normally limited in healthy brain tissue. However, collagens are upregulated to become an integral part of the ECM in certain forms of GBM (177, 285). Collagen expression has been shown to play a critical role in tumor immune suppression (354) and angiogenesis (284). Along with hyaluronic acid, collagen is a major contributor to matrix stiffness, a phenotype that has been shown to contribute to tumor aggressiveness (355). One study of patient microarray datasets found that six collagen genes (COL1A1, COL1A2, COL3A1, COL4A1, COL4A2, and COL5A2) play a role in immunosuppression and epithelial-mesenchymal transition in glioblastoma (356). Another study found that increased COL5A1 expression has been found to correlate strongly with lower survival probability in GBM patients (357). Additionally, differences in collagen organization may be a prognostic factor and these components represent potential therapeutic targets for glioblastoma treatment.

The receptors that interact with collagen are another important potential target. In a study of 29 GBM patients, mRNA levels of collagen receptor DDR1 were correlated with decreased survival (358), and its inhibition was shown to sensitize cells to both radio- and chemotherapy *in vitro (* 359 *)*. Receptor Endo180 binds to collagens I, IV, and V, and may be responsible for collagen remodeling in glioma (177). Chen et al. identified that type I collagen produced by pancreatic cancer cells is the abnormal oncogenic homotrimer variant and its deletion in cancer cells inhibit tumor progression and enriches T cells with enhanced efficacy of anti-PD1 immunotherapy (16). We recently demonstrated that inhibition of COL1A1 expression within the GBM tumor cells reprogramed the tumoral microenvironment and inhibited tumor invasion and progression (15).

## Summary and conclusions

A myriad of clinical trials for glioblastoma have been performed throughout the past decades, with no major successes. However, most of these have not focused on targeting multiple GBM mechanisms at once through combination therapies (360). Future research needs to consider the complexity of the GBM microenvironment, and tumor cell-ECM interactions, and use a multi-modal therapeutic approach, targeting both the cellular components and non-cellular components of the TME.

Glioblastoma patients have a high prevalence of tumor recurrence, signifying poor prognosis and rapid death (361). Key interactions between motile glioma cells and the non-cellular components of the TME stimulates rapid invasion of the primary tumor to other areas of the brain, forming deadly secondary tumors (3, 54, 346, 362). As GBM spreads throughout the brain, normal brain functions are further disrupted, and tumor lethality ensues. Aligned ECM components help glioma cells invade outwards into the normal brain in a rapid and persistent manner, forming organized structures called oncostreams (15). Targeting the basis of oncostream formation – collagen and fibronectin fibers – could eliminate these malignant structures, resulting in a less aggressive GBM tumor which may better respond to the traditional therapeutic approaches.

The ECM in the tumor hinders immune cell infiltration, as, unlike glioma cells, immune cells cannot reorganize the dense ECM and are blocked from attacking cancer cells in the tumor core (14, 363). Even those that infiltrate the tumor core have been shown to be exhausted (364). Targeting ECM components within the tumor can augment immune cell trafficking into the tumor (346), and studying exhaustion markers could reveal a strategy to revamp immune cell responses. Taking into consideration the high expression and the central role of the ECM components in glioma tumors growth and invasion, a chemotherapeutic approach using the ECM as a target should be of vital importance.

Additionally, because of these complex interactions between glioma cells and the non-cellular components in their microenvironment, researchers should consider using more accurate 3D or *ex-vivo* environments in their studies (15, 277, 289). Many results from 2D studies have been found to differ, sometimes even showing opposite results, compared to 3D studies that incorporate ECM components into the cellular environment (271–275). Glioma cells are highly sensitive to the biomechanical properties of their environment, including stiffness and viscoelasticity; modulating these properties to decrease their invasive strength and overall motility is a promising avenue for future research. We predict that future studies will concentrate on targeting the ECM directly, possibly by altering the tumoral production of ECM proteins - reducing tumor growth and improving the infiltration of immune cells - and thereby provide improved therapies for highly malignant brain tumors.

## Author contributions

SF, AC, MV, AA, EB, CA, MC, and PL wrote the manuscript, with overall guidance, revisions, and edits from PL and MC. SF prepared figures. SF and AA reviewed and edited the manuscript. All authors contributed to the article and approved the submitted version.

## Funding

This work was supported by National Institutes of Health/National Institute of Neurological Disorders and Stroke (NIH/NINDS) grants: R37-NS094804, R01-NS105556, R21-NS107894, R21-NS091555; R01-NS074387 to M.G.C.; National Institute of Neurological Disorders and Stroke (NIH/NINDS) grants: R01-NS076991, R01-NS096756, R01-NS082311, R01-NS122234, R01-NS127378 to P.R.L.; National Institute of Biomedical Imaging and Bioengineering (NIH/NIBI): R01-EB022563; National Cancer Institute (NIH/NCI) U01CA224160; Rogel Cancer Center at The University of Michigan G023089 to M.G.C. Ian’s Friends Foundation grant G024230, Leah’s Happy Hearts Foundation grant G013908, Pediatric Brain Tumor Foundation grant G023387 and ChadTough Foundation grant G023419 to P.R.L. RNA Biomedicine grant: F046166 to M.G.C. National Cancer Institute (NIH/NCI) grants: R01 CA125577 and R01 CA107469 to CGK. Health and Human Services, National Institutes of Health, UL1 TR002240 to Michigan Institute for Clinical and Health Research (MICHR), Postdoctoral Translational Scholars Program (PTSP), Project F049768 to A.C. National Science Foundation Graduate Research Fellowship Program to A.E.A (DGE #1841052).

## Acknowledgments

We thank all members of our laboratory for advice and comments on this work.

## Conflict of interest

The authors declare that the research was conducted in the absence of any commercial or financial relationships that could be construed as a potential conflict of interest.

## Publisher’s note

All claims expressed in this article are solely those of the authors and do not necessarily represent those of their affiliated organizations, or those of the publisher, the editors and the reviewers. Any product that may be evaluated in this article, or claim that may be made by its manufacturer, is not guaranteed or endorsed by the publisher.
